# Uncomplicated Monochorionic Twins: Two Normal Hearts Sharing One Placenta

**DOI:** 10.3390/jcm9113602

**Published:** 2020-11-09

**Authors:** Ximena Torres, Mar Bennasar, Laura García-Otero, Raigam J. Martínez-Portilla, Brenda Valenzuela-Alcaraz, Fátima Crispi, Anna Goncé, Eduard Gratacós, Francesc Figueras, Josep M. Martínez

**Affiliations:** 1 Fetal Medicine Research Center, BCNatal-Barcelona Center for Maternal-Fetal and Neonatal Medicine (Hospital Clínic and Hospital Sant Joan de Deu), 08028 Barcelona, Spain; lagarcia@clinic.cat (L.G.-O.); raifet@hotmail.com (R.J.M.-P.); valenzuela@clinic.cat (B.V.-A.); fcrispi@clinic.cat (F.C.); agonce@clinic.cat (A.G.); gratacos@clinic.cat (E.G.); ffiguera@clinic.cat (F.F.); jmmarti@clinic.cat (J.M.M.); 2 Faculty of Medicine, University of Barcelona, 08007 Barcelona, Spain; 3 Institut d’Investigacions Biomèdiques August Pi i Sunyer (IDIBAPS), 08036 Barcelona, Spain; 4 Centre for Biomedical Research on Rare Diseases (CIBER-ER), 08028 Barcelona, Spain

**Keywords:** uncomplicated monochorionic twins, fetal echocardiography, cardiac adaptation, cardiac function, cardiac shape, B-type natriuretic peptide

## Abstract

Cardiovascular dysfunction has been reported in complicated monochorionic diamniotic (MCDA) pregnancies; however, little is known whether hemodynamic changes occur in uncomplicated MCDA twins. A prospective observational study was conducted including 100 uncomplicated MCDA twins matched by gestational age to 200 low-risk singletons. Echocardiography was performed at 26–30 weeks gestation and cord blood B-type natriuretic peptide (BNP) was measured at delivery. In both groups, z-scores for echocardiographic parameters were within normal ranges; however the monochorionic group had larger atrial areas (mean (standard deviation) right atria-to-heart ratio: 17.0 (2) vs. 15.9 (1); *p* = 0.018; left atria-to-heart ratio: 17.0 (3) vs. 15.8 (2); *p* < 0.001) and signs of concentric hypertrophy (right relative wall thickness: 0.66 (0.12) vs. 0.56 (0.11); *p* < 0.001; left relative wall thickness: 0.69 (0.14) vs. 0.58 (0.12); *p* < 0.001). Longitudinal function was increased in twins, leading to higher tricuspid annular plane systolic excursion (6.9 mm (0.9) vs. 5.9 mm (0.7); *p* < 0.001) and mitral annular plane systolic excursion (4.9 mm (0.8) vs. 4.4 mm (1.1); *p* < 0.001. BNP levels at birth were also higher in MCDA twins (median [interquartile range]: 20.81 pg/mL [16.69–34.01] vs. 13.14 pg/mL [9.17–19.84]; *p* < 0.001). Thus, uncomplicated MCDA fetuses have normal cardiac shape and function, but signs of cardiac adaptation were identified by echocardiographic and biochemical parameters, when compared with singletons.

## 1. Introduction

Monochorionic diamniotic (MCDA) twin pregnancies are characterized by a unique placenta in which two vascular territories are connected by vascular anastomoses, leading to continuous and balanced intertwin transfusion [1,2]. Placental vascular architecture is known to influence fetal circulation and heart development in singleton pregnancies [3,4], and there are data indicating that monochorionic placentation may play a role in long-term cardiac functional patterns of children from uncomplicated MCDA twin pregnancies [5]. Normal hearts tend to adapt to volume and pressure loading patterns, which change throughout gestation and are influenced by the maturation stage of structures such as the placenta vascular bed and by the resistance of the circulatory system [6]. This physiologic cardiac adaptation preserves or enhances cardiac function and differs from pathologic cardiac remodeling in which cardiac contractile function eventually declines [7].

Cardiovascular changes secondary to imbalanced transfusion have been described in twin-to-twin transfusion syndrome (TTTS) [8] and selective fetal growth restriction (sFGR) [9] using Doppler analysis [10,11], echocardiographic parameters [12,13], and cardiac dysfunction biomarkers, such as fetal B-type natriuretic peptide (BNP) [14,15]. However, cardiac adaptation to continuous balanced transfusion between uncomplicated MCDA twin fetuses has not been integrally evaluated in prenatal life.

Therefore, the objective of this study was to compare functional and structural cardiac parameters of uncomplicated MCDA twins with those of low-risk singletons using fetal echocardiography. Umbilical cord plasma BNP levels were evaluated in a subset of neonates as well.

## 2. Materials and Methods

### 2.1. Study Protocol

In this prospective observational study, consecutive MCDA twins were matched to singletons in a 1:2 ratio by gestational age (GA) at the time of fetal ultrasound. Patients were recruited from January 2015 to November 2017 in the BCNatal Maternal-Fetal Medicine Department (Hospital Clinic and Hospital Sant Joan de Déu, Barcelona). Study exclusion criteria were (a) fetal growth restriction; (b) pregnancy conceived by assisted reproductive techniques; (c) prenatal complication, such as preterm delivery (< 35 weeks); (d) maternal complications, such as gestational diabetes mellitus, preeclampsia, or infection; (e) fetal malformation or chromosomal abnormality; (e) for MCDA twin pregnancies, additional exclusion criteria were the presence of TTTS, sFGR, twin anemia polycythemia syndrome, or fetal weight discordance >20%.

Maternal and gestational characteristics were collected. Perinatal data, including GA at delivery, mode of delivery, birthweight, Apgar scores, and umbilical artery pH, were also collected. GA was based on the crown-rump length (CRL) [16] on a standard 11–14-week ultrasound, with CRL of the larger twin used for MCDA twin pregnancies [17]. At delivery, venous cord blood was obtained for plasma BNP analysis. Ethics approval was obtained from our local ethics board (Code 2015/0484), and all participants provided written informed consent.

### 2.2. Fetal Ultrasound Evaluation

Fetal ultrasound scans were performed at 26–30 weeks’ gestation as part of our prenatal management protocol in MCDA pregnancies, using a Siemens Sonoline Antares device (Siemens Medical Systems, Erlangen, Germany), with a 4–6 MHz curved array transducer. Basic ultrasound evaluation included a detailed anatomical examination, as well as determination of the estimated fetal weight (EFW), fetal weight centile, and amniotic fluid. EFW was calculated using the Hadlock formula [18], and centiles were based on prescriptive curves for singleton [19] and MCDA twin fetuses [20], respectively. Monochorionicity was always confirmed by the presence of a T sign with a single placental mass [21] during the first trimester scan. Conventional fetoplacental Doppler assessment included umbilical artery (UA), middle cerebral artery (MCA), and ductus venosus (DV) pulsatility indices, as well as MCA peak systolic velocity (PSV), in accordance with previously published methodology [22,23,24,25].

Fetal echocardiography included comprehensive structural and functional cardiac evaluation. Structural measurements were performed according to standardized methodology recently described in a low-risk singleton population [26,27]. Cardiac morphometry included the cardiac, thoracic, ventricular, and atrial areas and the ventricular diameters. These measurements were obtained on two-dimensional images from an apical or basal four-chamber view at end-diastole, except for the atrial areas, which were determined at end-systole. Cardiothoracic ratio was calculated as cardiac area divided by thoracic area. Atria-to-heart and ventricle-to-heart ratios were calculated (atrial area/cardiac area) and (ventricular area/cardiac area), respectively. Ventricular sphericity indices were calculated as ventricular longitudinal diameter/basal diameter. A transverse four-chamber view was used to measure myocardial wall thickness (septum, left and right ventricular free walls), using M-mode at end-diastole and end-systole. Left and right relative wall thicknesses (RWTs) were calculated as (septal wall thickness + free wall thickness)/ventricular transverse diameter [27,28].

Cardiac functional evaluation included assessment of diastolic and systolic parameters. Atrioventricular flows were evaluated just below the mitral or tricuspid valve leaflets. Left and right E/A ratios were estimated by calculating the ratio between the E-wave, which is the passive ventricular filling phase, and the A-wave, which corresponds to the active ventricular filling phase [29]. Isovolumetric contraction time (ICT) and isovolumetric relaxation time (IRT) were determined by measuring the intervals from the closing of the mitral valve to the opening of the aortic valve and from the closing of the aortic valve to the opening of the mitral valve, respectively. Ejection time (ET) was the time between the opening and closing of the semilunar aortic valve. Left myocardial performance index (MPI) was calculated as (ICT + IRT)/ET [30]. Mitral annular plane systolic excursion (MAPSE) and tricuspid annular plane systolic excursion (TAPSE) were assessed by apical or basal four-chamber view in M-mode; the cursor was placed at a right angle to the atrioventricular junction, where the maximum amplitude of displacement was measured [31]. Shortening fraction and ejection fraction were also determined by M-mode, placing the cursor perpendicular to the septal wall at the atrioventricular valve level in the transverse four-chamber view. Maximum and minimum diameters corresponded to end-diastolic dimension (EDD) and end-systolic dimension (ESD), respectively. Shortening fraction was calculated as (EDD − ESD)/EDD, while ejection fraction was calculated as (EDD^3^ − ESD^3^)/EDD^3^ [32]. Left and right cardiac outputs (COs) were estimated as π × (aortic or pulmonary diameter/2)^2^ × (aortic or pulmonary artery systolic velocity-time integral (VTI)) × heart rate. Combined CO was calculated by adding the left and right CO values [33]. Aortic and pulmonary systolic VTIs were obtained by placing the caliper above the aortic or main pulmonary artery valve and manually tracing the spectral Doppler area under the curve. Left and right outflow tract diameters were measured during end-systole in the five-chamber and three-vessel tracheal views, respectively.

### 2.3. Cord Blood Sampling and B-Type Natriuretic Peptide Assessment

Umbilical vein cord blood samples were preserved, in a subgroup of cases, after cord clamping at delivery, promptly centrifugated at 3000 rpm for 10 min at 4 °C, and then stored at −80 °C until assayed. Plasma BNP levels were measured using the Siemens ADVIA Centaur^®^ BNP assay.

### 2.4. Statistical Analysis

Data were analyzed using MLwiN version 2.36 [34] (Centre for Multilevel Modelling, University of Bristol, Bristol, U.K.). Sample size was calculated by expecting at least a 0.03 difference in MPI between the MCDA twin and singleton groups, based on previous results of a 0.03 MPI difference between uncomplicated monochorionic twins and TTTS donors [12]. For a power of 80% and an alpha error of 0.05, a minimum of 83 fetuses in the MCDA twin group and 166 in the singleton group would be required (STATA Statistics/data analysis, version 15.1). To allow for drop-outs, the cohort finally included 50 uncomplicated MCDA twins (100 fetuses) and 200 singleton pregnancies.

Results are presented as mean (standard deviation), median (interquartile range (IQR)), or percentage. Differences were considered significant when *p* < 0.05. Maternal baseline and perinatal characteristics were compared between groups using Student’s *t*-test, Mann-Whitney U test, or Pearson’s chi-square test. Subsequently, multilevel analyses of echocardiographic parameters were adjusted for potential baseline confounders detected in the univariate analysis: chorionicity, EFW at time of ultrasound, and maternal smoking status. BNP multilevel analysis was adjusted for perinatal characteristics, such as chorionicity, smoking, GA at birth, delivery mode, and birthweight. Cardiac parameters for MCDA twins and singletons were normalized to z-scores, using formulas previously published and verified [24,25,26,27,29,30,31,32,33,35]. A one-way ANOVA with Bonferroni correction was performed to compare the echocardiographic parameter means of the larger and smaller twin, as well as the mean of the singleton group, and to evaluate possible selection bias, we performed a sensitivity analysis in fetuses with or without BNP measurements to assess differences in demographics, echocardiographic findings, or perinatal outcomes between these two groups. In addition, a Pearson correlation was performed to associate the BNP plasma birth levels and different echocardiographic parameters.

## 3. Results

### 3.1. Baseline and Perinatal Data

**Figure 1:**
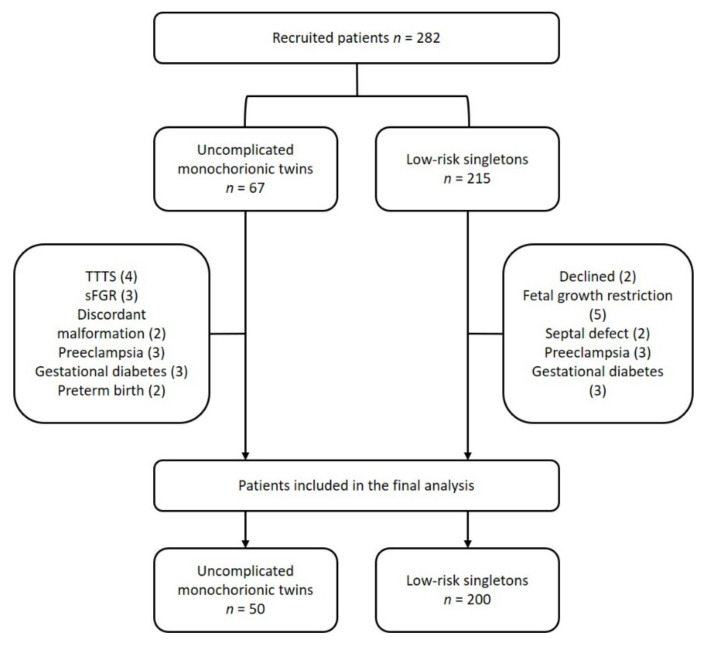
Recruitment flow chart. Fetal echocardiography data were collected from all patients who agreed to participate in the study. Some patients were excluded from further analysis because birthweight and perinatal outcomes met the exclusion criteria. TTTS: twin-to-twin transfusion syndrome, sFGR: selective fetal growth restriction.

Figure 1 represents the flow diagram of the selected cohort and reasons for exclusion.

Maternal baseline characteristics and perinatal outcomes of the 100 uncomplicated MCDA twin fetuses and 200 GA-matched singletons are shown in Table 1.

There were no differences in maternal age, parity, body mass index, socioeconomic status, or ethnicity between groups. However, smoking was less prevalent in the MCDA twin group than in the singleton group. There were no differences in GA at the time of cardiac ultrasound examination. As expected, singletons were heavier than MCDA twin fetuses, and delivery occurred later in pregnancy, with a lower caesarean-section rate. In addition, there were not significant differences between in the monochorionic pair in terms birthweight discordancy (mean 6.7%; SD 5.6). Perinatal data, including 5 min Apgar score and umbilical artery pH, did not differ significantly between the monochorionic and the singleton group.

### 3.2. Echocardiography

Echocardiographic results are shown in Table 2.

All comparisons between groups were performed after adjusting for EFW, smoking, and chorionicity. Cardiac size was not significantly different between the two groups, but there were other cardiac structural differences. In particular, MCDA twin fetuses had larger right and left atrial areas and smaller right and left ventricular areas and dimensions.

These results were supported by the relative size assessments, as MCDA twins had higher atria-to-heart ratios bilaterally and lower ventricle-to-heart ratios bilaterally, when compared to singleton fetuses. Moreover, MCDA twin fetuses had thicker ventricular walls in both systole and diastole at the expense of more globular ventricular cavities, as shown by lower sphericity indices and higher relative wall thicknesses bilaterally.

Cardiac function during both systole and diastole also differed between MCDA twins and singletons (Table 2). The MCDA twin group had a higher DV pulsatility index, higher percentage of patients with tricuspid regurgitation, and longer isovolumetric relaxation time than the singleton group. MCDA twins also exhibited increased right and left longitudinal function, represented by higher TAPSE and MAPSE values, and increased right and left radial motion, demonstrated by higher ejection fraction, shortening fraction, and cardiac output.

### 3.3. Echocardiography z-Scores

Table 3 shows the mean values of cardiac parameters transformed into z-scores.

**Figure 2:**
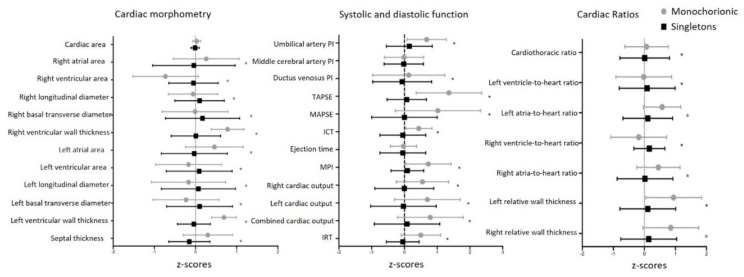
Forest plots for z-scores (mean and standard deviation) of cardiac morphometric parameters, functional systolic parameters and functional diastolic parameters and cardiac ratios. * Statistically significant results. PI: pulsatility index; TAPSE: tricuspid annular plane systolic excursion; MAPSE: mitral annular plane systolic excursion; ICT: isovolumetric contraction time; IRT: isovolumetric relaxation time.

When comparing z-scores, differences in cardiac systolic and morphometric parameters, as well as isovolumetric relaxation time, persisted. These results are also shown in Figure 2.

**Figure 3:**
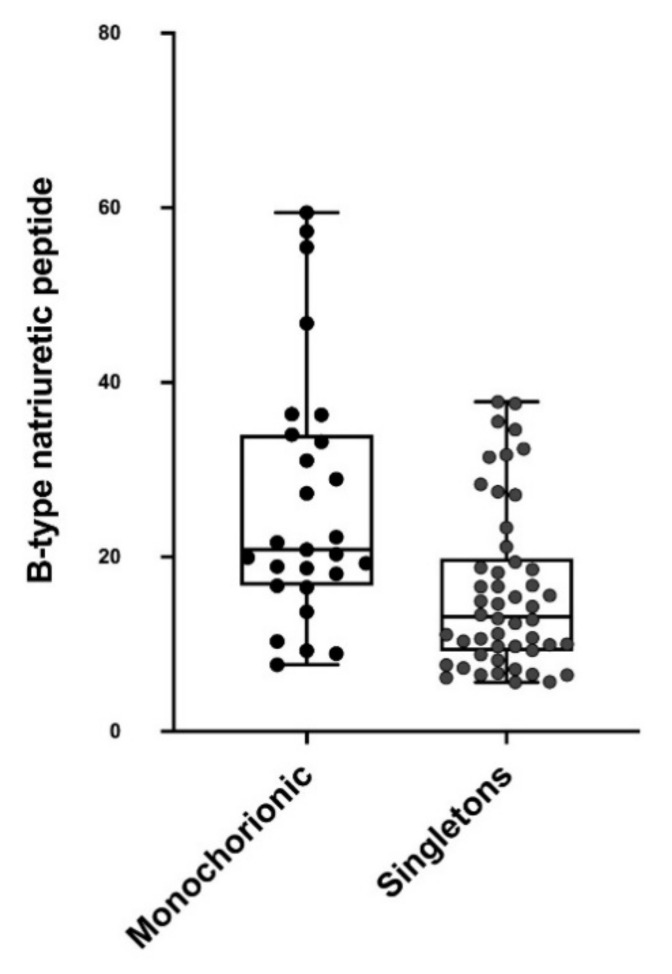
Bee swarm plot showing the median and distribution of plasma B-type natriuretic peptide (BNP) levels at birth of 27 uncomplicated monochorionic diamniotic twin fetuses and 50 low-risk singletons. *p* < 0.001 by multilevel analysis adjusted for chorionicity, birthweight, smoking, gestational age at delivery, and delivery mode.

Cord blood subgroup samples for BNP measurements in 27 MCDA twins and 50 singletons were analyzed. The MCDA twin group had a significantly higher median (IQR) plasma BNP concentration than singletons (20.81 pg/mL [16.69–34.01] vs. 13.14 pg/mL [9.17–19.84]; *p* < 0.001) (Figure 3).

A supplementary table (Table S1) is included as supplemental material in which we provide the echocardiographic parameter means of the larger and smaller twin, as well as the mean of the singleton group, with no significant differences within the twins. Sensitivity analyses of patients with or without BNP measurements (to assess potential selection bias) revealed no significant differences in baseline characteristics, echocardiographic findings, or perinatal outcomes between patients who did or did not undergo BNP analysis. Most representative echocardiographic parameters of the sensitivity analysis are showed in the Table S2 in the Supplementary Material.

**Figure 4:**
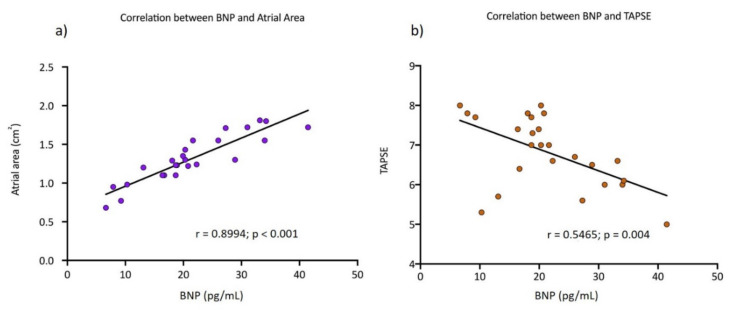
(**a**) Scatter plot exhibits the positive linear correlation between the atrial area (cm^2^) and the BNP plasma levels (pg/mL) (r = 0.8994, *p* < 0.001). (**b**) Scatter plot shows the negative correlation between the tricuspid annular plane systolic excursion (TAPSE, mm) and the BNP plasma levels (pg/mL) (r = 0.5465, *p* = 0.004).

We performed a Pearson correlation and the strongest association was between BNP birth levels and the right auricular area at the ultrasound (r = 0.8994, *p* < 0.001) Moreover, an acceptable association was found between TAPSE and BNP (r = 0.5465, *p* = 0.004). These correlations are shown in Figure 4.

## 4. Discussion

This study provides evidence that heart shape and function are within normal ranges in uncomplicated MCDA twin fetuses, according to echocardiographic parameters. However, some differences are present, compared with singleton fetuses. Cardiac size was similar between the two groups of fetuses, but MCDA twins had dilated atria in terms of size adjusted by EFW and relative size (atria-to-heart ratio), as well as smaller and more spherical ventricles with thickened walls. Together with these structural changes, cardiac function adaptation results in a more efficient myocardium with improved systolic function; nevertheless, signs of subclinical diastolic dysfunction are present as well, as evidenced by increased levels of plasma BNP at birth.

Our results are consistent with the limited published data of cardiovascular physiology in uncomplicated MCDA twin fetuses. MPI is a parameter frequently used to evaluate global cardiac function, either in singleton or MCDA twin fetuses [8,12,36]. Zanardini et al. [37] recently published reference MPI charts for up to 26 weeks’ gestation, and Ortiz et al. [12] compared MPI and time intervals of 94 uncomplicated monochorionic twins and TTTS twins at 20 weeks’ gestation. Despite differences in GA at echocardiographic assessment, our results are consistent with data from these studies (with similar mean values in the uncomplicated MCDA twins’ group), confirming that MPI and time intervals do not vary significantly during pregnancy in uncomplicated MCDA pregnancies. However, when comparing to singleton pregnancies, the MCDA group had higher MPI at the expense of the longer isovolumetric contraction and relaxation times that might be generated by the ventricular hypertrophy observed in this group.

Cardiac output has also been assessed in uncomplicated MCDA twin fetuses. In a longitudinal evaluation, Sueters et al. [38] compared median cardiac output/kg in 20 non-TTTS monochorionic twins (10 donors and 10 recipients), which was significantly higher than that in singletons (*p* < 0.001). This is consistent with our findings of higher cardiac output in non-complicated MCDA twins. Thus, our findings are in line with reports showing a higher incidence of tricuspid regurgitation, increased DV pulsatility index, and discordant nuchal translucency in euploid MCDA pregnancies [39,40], suggesting that cardiac overload may begin in the first trimester associated with decreased compliance.

Longitudinal motion has been previously evaluated in MCDA twins; Ortiz et al. [13] and Wohlmuth et al. [8] compared MAPSE and TAPSE in fetuses with TTTS before and after laser therapy with controls at 21 weeks’ gestation; however, these results cannot be compared to ours because longitudinal parameters change over time, and our study evaluated fetuses at an older GA than that in previous studies.

Cardiac function can also be evaluated by means of Doppler parameters. A recent publication of longitudinal Doppler reference ranges in MCDA twin fetuses [41] reported increased median UA pulsatility index values, compared with values in reference singleton nomograms, reflecting the higher cardiac afterload of monochorionic placentation and continuous intertwin blood exchange. These results are consistent with the higher UA pulsatility index z-score observed in our MCDA twins. Our results also showed significantly higher mean DV pulsatility index in MCDA twins than in singletons. Although previous studies reported similar median DV pulsatility index in singletons and MCDA pregnancies, the upper centiles were higher in MCDA twins.

Regarding morphometric evaluation, cardiothoracic ratio is the most studied parameter in MCDA twins. We found no difference in cardiothoracic ratio between groups of fetuses. Although no specific reference values have been reported for MCDA twin pregnancies, Sueters et al. [42] reported similar cardiothoracic ratios between singletons and non-TTTS, which was in accordance with our results. In a prospective study, Karatza et al. [43] compared 89 uncomplicated MCDA pairs of twins with TTTS fetuses at 24 weeks gestation and found that the mean cardiothoracic ratio in uncomplicated MCDA twins was slightly lower than the mean in our MCDA twins. This difference was likely due to differences in GAs at the time of analysis.

There are no reference ranges for plasma BNP levels in uncomplicated MCDA twin newborns. However, our BNP median concentration was slightly lower than values at birth reported by Bajoria et al. [44] in uncomplicated MCDA twins and significantly higher than the median BNP in our singleton group. Moreover, median birth BNP concentration in our low-risk singletons was within the normal range for healthy newborns reported by Rodriguez et al. [45] (median 12.15 pg/mL [IQR of 7.7–16.8]). The higher BNP results in MCDA twin pregnancies have a strong correlation with the increased atrial area, resulting from a higher volume of blood reaching the atria and leading to atrial wall distension [46,47]. These findings enhance our conclusion about cardiac adaptation to volume in monochorionic hearts; thus, in monochorionic twin fetuses at the same gestational age without significant differences in weight discordance with their co-twin and between the singleton group, higher birth BNP levels are present in fetuses with larger atrial areas and lower TAPSE (Figure 4). The importance of these statistical correlations is that presumably we can identify a subgroup of monochorionic patients who will be candidates for a stricter cardiovascular follow-up in infancy. However, these findings must be confirmed and correlated with postnatal echocardiography.

From a pathophysiological perspective, cardiac hypertrophy associated with atrial distension is the usual response to a pressure and volume overload. In our series, volume overload had an impact on the dilated atrial size consistent with higher BNP levels at birth, adaptative changes that are linked to the higher DV pulsatility index, the increased bilateral velocity time integral that exhibit the amount of blood ejected per heartbeat, and consequently the cardiac output. Furthermore, a concentric hypertrophy pattern was present in MCDA twins, probably reflecting the direct effect of increased afterload imposed by the monochorionic placenta. It has been hypothesized that in all monochorionic twins, a large of number of bidirectional arteriovenous connections are present in early pregnancy, but progressive spontaneous closure or disruption of these anastomoses develops with advancing gestation, a process that occurs at random. When the reduction in anastomoses is asymmetrical, TTTS may be triggered [48]. However, in uncomplicated MCDA twins, a well-balanced reduction in vessels increases cardiac afterload. This increased placental resistance is also consistent with increased UA pulsatility index values published in recent monochorionic Doppler references [41] and with our findings of increased UA pulsatility index z-score.

The structural changes in our MCDA twins were associated with enhanced systolic function, as reflected by higher longitudinal motion, ejection fraction, and cardiac output values. The improvements in systolic parameters and absence of ventricular dilatation indicate that the hearts of MCDA twins underwent physiological adaptation to volume overload. This is consistent with the fact that end-diastolic volume regulates work of the heart in the early stages of cardiac compensation, improving ventricular contraction and volume of blood ejected by the heart, according to the Frank-Starling model [49].

### Strengths and Limitations

As a major strength, this is the largest cohort of MCDA twin pregnancies and low-risk singletons prospectively evaluated by experienced fetomaternal specialists at two referral centers in which patients received conventional ultrasound assessment until delivery. This entails that monochorionic pregnancies were followed every 2 weeks. Therefore, significant weight discordance (> 20%); amniotic fluid discordance; or TTTS, sFGR, and TAPS criteria were excluded from the analysis and referred to a high-risk monochorionic unit. Other strengths are the stringent selection criteria and the multilevel analyses allowing us to model and compare variances among different levels of exposure; this statistical technique is the best way to assess hierarchical data, considering the variance of each level. Moreover, the two-step analysis performed at 28 weeks and birth allowed us to detect differences between fetal hearts exposed to the in utero environment sufficiently long to produce cardiac changes and, subsequently, relate these findings to plasma BNP levels at birth.

Potential study limitations should be acknowledged. The use of low-risk singletons to compare monochorionic cardiac function and structure may prove controversial. However, the lack of dichorionic cardiac reference values prevents a standardized quantitative assessment of the measurements using z-scores. Moreover, we performed comprehensive echocardiography where some parameters (e.g., cardiac output, ejection fraction, shortening fraction) may be technically difficult to reproduce. Nevertheless, we optimized technical variations to minimize errors by having the same group of experienced operators perform all ultrasound scans and using mean values of three measurements of three consecutive cardiac cycles. Moreover, the same operators recently demonstrated good reproducibility in structural echocardiographic parameters in singletons through 2D imaging and M mode [26,27,50]. Furthermore, cord blood samples were obtained from only some of our newborns; however, work-up bias was unlikely, as there were no differences in baseline, echocardiographic, or perinatal outcomes between patients with or without BNP samples.

## 5. Conclusions

In conclusion, since uncomplicated MCDA twin fetuses have echocardiographically normal cardiac shape and function and given the lack of monochorionic specific cardiac reference charts, singletons’ nomograms can be used in clinical practice. However, when compared with low-risk singletons, they exhibit signs of cardiac hypertrophy and atrial dilatation that can be explained by in utero volume and pressure overloads, which lead to functional changes of enhanced systolic function and subclinical diastolic impairment. Whether these findings can be used to improve the management of some complicated monochorionic cases, for example, to determine a surgical approach in cases of twin-to-twin transfusion syndrome stage I with cardiac dysfunction or to indicate the termination of pregnancy in sFGR with cardiovascular abnormalities, deserves further evaluation. Certainly, these conclusions open future opportunities for short- and long-term research about postnatal consequences of these cardiovascular adaptations.

## Figures and Tables

**Table 1:** Maternal and perinatal characteristics of 100 monochorionic diamniotic twin fetuses and 200 low-risk singletons.

Characteristic	MCDA Fetuses (*n* = 100)	Singleton (*n* = 200)	*p*
Maternal			
Age (years)	33 (4)	32 (4)	0.406
Height (m)	1.63 (0.1)	1.63 (0.1)	0.225
Weight (kg)	60.9 (6.9)	61.3 (7.4)	0.079
Body mass index (kg/m^2^)	23.2 (2.5)	23.8 (3.2)	0.110
Caucasian ethnicity	83%	81%	0.105
Smoking during pregnancy	2%	9%	0.002
Low socioeconomic status	6%	11%	0.162
Primiparous	55%	61%	0.221
Perinatal data			
Gestational age at birth (weeks)	36.3 (1.1)	39.5 (1.6)	<0.001
Birthweight (g)	2395 (380)	3255 (477)	<0.001
Male sex	42%	50%	0.192
Cesarean section	65%	15%	<0.001
Apgar at 5 min	9 [7–10]	9 [7–10]	0.498
Umbilical artery pH	7.22 [7.16–7.28]	7.21 [7.15–7.26]	0.450

Data are mean (SD), median [interquartile range], or percentage, as appropriate. MCDA: monochorionic diamniotic.

**Table 2:** Cardiac functional and structural parameters of 100 monochorionic diamniotic twin fetuses and 200 low-risk singletons.

Characteristic	MCDA Fetuses (*n* = 100)	Singleton (*n* = 200)	*p* *
Gestational age at ultrasound (weeks)	28.0 (2.2)	28.0 (2.3)	0.862
Standard fetoplacental data			
Estimated fetal weight at ultrasound (g)	1122 (240)	1213 (262)	0.027
Estimated fetal weight centile	49 (24)	55 (28)	0.059
Umbilical artery PI	1.10 (0.2)	1.03 (0.2)	0.191
Middle cerebral artery PI	1.84 (0.2)	1.82 (0.3)	0.743
Ductus venosus PI	0.60 (0.2)	0.53 (0.1)	0.007
Heart rate			
Left fetal heart rate (beats/min)	140 (8)	141 (7)	0.092
Right fetal heart rate (beats/min)	141 (7)	141 (7)	0.102
Cardiac morphometry			
Aortic diameter (mm)	4.7 (0.6)	4.5 (0.7)	0.201
Main pulmonary diameter (mm)	5.0 (0.7)	4.8 (0.8)	0.110
Cardiac area (cm^2^)	8.1 (1.7)	8.0 (4.5)	0.554
Thorax area (cm^2^)	30 (6)	31 (6)	0.217
Cardiothoracic ratio	0.26 (0.02)	0.26 (0.03)	0.923
Right atrial area (cm^2^)	1.36 (0.4)	1.24 (0.4)	0.001
Right atria-to-heart ratio	0.17 (0.02)	0.15 (0.01)	0.018
Right ventricular area (cm^2^)	1.74 (0.5)	1.85 (0.4)	0.001
Right ventricle-to-heart ratio	0.21 (0.04)	0.24 (0.03)	0.001
Left atrial area (cm^2^)	1.37 (0.3)	1.21 (0.3)	0.001
Left atria-to-heart ratio	0.17 (0.03)	0.15 (0.02)	<0.001
Left ventricular area (cm^2^)	1.87 (0.5)	1.99 (0.5)	0.001
Left ventricle-to-heart ratio	0.24 (0.04)	0.26 (0.04)	0.019
Right longitudinal diameter (mm)	17.1 (2.0)	17.6 (2.1)	0.001
Right basal transverse diameter (mm)	9.8 (1.3)	10.2 (1)	0.001
Right ventricular sphericity index	1.68 (0.2)	1.73 (0.2)	0.021
Left longitudinal diameter (mm)	19.6 (3)	19.9 (3)	0.001
Left basal transverse diameter (mm)	10.6 (2)	10.5 (2)	0.001
Left ventricular sphericity index	1.86 (0.2)	1.90 (0.2)	0.045
Right free diastolic wall thickness (mm)	2.9 (0.3)	2.6 (0.3)	<0.001
Left free diastolic wall thickness (mm)	2.9 (0.3)	2.6 (0.3)	<0.001
Septal diastolic wall thickness (mm)	3.0 (0.4)	2.8 (0.3)	0.001
Right relative wall thickness (mm)	0.66 (0.12)	0.56 (0.11)	<0.001
Left relative wall thickness (mm)	0.69 (0.14)	0.58 (0.12)	<0.001
Systolic function			
Aortic VTI (cm^2^)	9.7 (1.7)	8.1 (1.0)	0.001
Main pulmonary VTI (cm^2^)	9.2 (1.5)	8.0 (1.0)	0.001
Tricuspid annular plane systolic excursion (mm)	6.9 (0.9)	5.9 (0.7)	<0.001
Mitral annular plane systolic excursion (mm)	4.9 (0.8)	4.4 (1.1)	<0.001
Isovolumetric contraction time (ms)	32 (4)	29 (3)	0.014
Ejection time (ms)	172 (11)	170 (12)	0.242
Myocardial performance index	0.44 (0.05)	0.40 (0.04)	0.031
Right stroke volume (mL)	1.82 (0.6)	1.58 (0.6)	0.001
Right cardiac output (mL/min)	257 (87)	221 (87)	<0.001
Left stroke volume (mL)	1.66 (0.6)	1.35 (0.5)	<0.001
Left cardiac output (mL/min)	234 (87)	189 (69)	<0.001
Combined cardiac output (mL/min)	492 (160)	411 (145)	<0.001
Right ejection fraction (%)	77.8 (9.3)	67.2 (11.3)	0.002
Right shortening fraction	41.9 (9.1)	34.4 (8.9)	0.003
Left ejection fraction (%)	80.5 (8.6)	73.7 (11.2)	0.023
Left shortening fraction	42.9 (9.1)	37.2 (9.9)	0.015
Diastolic function			
Tricuspid E (cm/seg)	37 (8)	34 (7)	0.061
Tricuspid A (cm/seg)	52 (10)	50 (9)	0.055
Tricuspid E/A ratio	0.71 (0.1)	0.70 (0.1)	0.064
Mitral E (cm/seg)	34 (8)	32 (6)	0.067
Mitral A (cm/seg)	48 (9)	47 (8)	0.108
Mitral E/A ratio	0.71 (0.1)	0.68 (0.1)	0.190
Isovolumetric relaxation time (ms)	43 (5)	39 (6)	0.031
Tricuspid regurgitation (%)	13	6	<0.001

Data are mean (SD) or percentage, as appropriate. * *p*-value adjusted for smoking, estimated fetal weight at time of ultrasound, and chorionicity. MCDA: monochorionic diamniotic; PI: pulsatility index, VTI: velocity-time integral.

**Table 3:** Doppler, cardiac morphometric, and functional parameter z-scores of 100 monochorionic diamniotic twin fetuses and 200 low-risk singletons.

Characteristic	MCDA Fetuses (*n* = 100)	Singleton (*n* = 200)	*p*
Doppler			
Umbilical artery PI	0.68 (0.6)	0.15 (0.7)	0.042
Middle cerebral artery PI	−0.01 (0.6)	−0.02 (0.6)	0.944
Ductus venosus PI	0.13 (1.1)	−0.07 (0.9)	0.021
Cardiac morphometry			
Cardiac area	0.03 (0.1)	−0.01 (0.1)	0.579
Cardiothoracic ratio	0.07 (0.7)	0.01 (0.8)	0.923
Left atrial area	0.46 (0.7)	−0.03 (0.8)	0.001
Left atria-to-heart ratio	0.57 (0.6)	0.11 (0.8)	0.001
Left ventricular area	−0.17 (0.8)	0.09 (0.8)	0.019
Left ventricle-to-heart ratio	−0.02 (0.9)	0.09 (0.9)	0.023
Right atrial area	0.26 (0.8)	−0.04 (1.0)	0.008
Right atria-to-heart ratio	0.45 (0.7)	0.02 (0.9)	0.001
Right ventricular area	−0.73 (0.8)	−0.05 (0.6)	0.001
Right ventricle-to-heart ratio	−0.18 (0.9)	0.16 (0.5)	0.001
Right longitudinal diameter	−0.06 (0.6)	0.10 (0.6)	0.046
Right basal transverse diameter	−0.01 (0.8)	0.17 (0.9)	0.001
Right ventricular sphericity index	−0.08 (0.3)	0.05 (0.5)	0.036
Left longitudinal diameter	−0.17 (0.9)	0.07 (0.9)	0.047
Left basal transverse diameter	−0.23 (0.8)	−0.10 (0.8)	0.020
Left ventricular sphericity index	−0.14 (0.7)	0.05 (0.7)	0.045
Right ventricular diastolic wall thickness	0.78 (0.4)	0.01 (0.6)	0.001
Left ventricular diastolic wall thickness	0.69 (0.3)	−0.04 (0.4)	0.001
Septal diastolic thickness	0.30 (0.6)	−0.15 (0.5)	0.001
Right relative wall thickness	0.85 (0.9)	0.14 (0.9)	0.001
Left relative wall thickness	0.94 (0.9)	0.11 (0.9)	0.001
Systolic function			
Tricuspid annular plane systolic excursion	1.36 (1.0)	0.07 (0.6)	0.001
Mitral annular plane systolic excursion	1.02 (1.3)	0.00 (1.0)	0.001
Isovolumetric contraction time	0.44 (0.4)	0.06 (0.4)	0.009
Ejection time	−0.03 (0.4)	−0.05 (0.7)	0.762
Myocardial performance index	0.73 (0.7)	0.09 (0.5)	0.008
Right cardiac output	0.55 (0.8)	−0.03 (0.9)	0.009
Left cardiac output	0.70 (1.0)	−0,03 (1.0)	0.045
Combined cardiac output	0.79 (1.0)	0.08 (1.0)	0.001
Diastolic function			
Isovolumetric relaxation time	0.50 (0.6)	−0.05 (0.5)	0.001

Data are mean (SD). MCDA: monochorionic diamniotic; PI: pulsatility index.

## Supplementary Materials

The following are available online at https://www.mdpi.com/2077-0383/9/11/3602/s1, Table S1: Cardiac functional and structural parameters of 50 monochorionic diamniotic larger twins, 50 monochorionic smaller twins and 200 low-risk singletons, Table S2: Sensitivity analysis of the monochorionic and the singleton groups with and without BNP analysis.
