# Supplementary material for: Uncomplicated Monochorionic Twins: Two Normal Hearts Sharing One Placenta
Source: J Clin Med. 2020 Nov 9;9(11):3602. doi: 10.3390/jcm9113602 (PMC7695261; doi:10.3390/jcm9113602)
Supplement: Supplementary file 1 [file jcm-09-03602-s001.pdf]

Supplementary Table S1. Cardiac functional and structural parameters of 50 monochorionic diamniotic larger twins, 50 monochorionic smaller twins and 200 low-risk singletons

Characteristic	MCDA larger twins⁺ (*n*=50)	MCDA smaller⁺ twins (*n*=50)	Singleton (*n*=200)	*p**
Gestational age at ultrasound (weeks)	28.0(1.6)	28.0 (1.6))	28.0 (2.3)	0.672
Standard fetoplacental data				
Estimated fetal weight at ultrasound (g)	1175 (320)	1115 (240)	1213 (262)	0.033
Estimated fetal weight centile	52 (26)	47 (24)	55 (28)	0.078
Umbilical artery PI	1.08 (0.2)	1.11 (0.2)	1.03 (0.2)	0.122
Middle cerebral artery PI	1.85 (0.2)	1.83 (0.2)	1.82 (0.3)	0.618
Ductus venosus PI	0.59 (0.2)	0.61 (0.2)	0.53 (0.1)	0.007
Heart rate				
Left fetal heart rate (beats/min)	141 (9)	140 (8)	141 (7)	0.379
Right fetal heart rate (beats/min)	141 (6)	141 (7)	141 (7)	0.826
Cardiac morphometry				
Aortic diameter (mm)	4.75 (0.5)	4.64 (0.6)	4.5 (0.7)	0.228
Main pulmonary diameter (mm)	5.1 (0.7)	4.9 (0.5)	4.8 (0.8)	0.117
Cardiac area (cm^2)	8.1 (1.8)	7.9 (1.7)	8.0 (4.5)	0.112
Thorax area (cm^2)	31 (7)	29.3 (6)	31 (6)	0.564
Cardiothoracic ratio	0.26 (0.03)	0.26 (0.02)	0.26 (0.03)	0.734
Right atrial area (cm^2)	1.35 (0.4)	1.36 (0.4)	1.24 (0.4)	0.008
Right atria-to-heart ratio	0.16 (0.02)	0.17 (0.02)	0.15 (0.01)	0.013
Right ventricular area (cm^2)	1.75(0.6)	1.73 (0.5)	1.85 (0.4)	0.001
Right ventricle-to-heart ratio	0.21 (0.04)	0.20 (0.04)	0.24 (0.03)	0.015
Left atrial area (cm^2)	1.35 (0.3)	1.37 (0.3)	1.21 (0.3)	0.001
Left atria-to-heart ratio	0.16 (0.02)	0.17 (0.03)	0.15 (0.02)	0.001
Left ventricular area (cm^2)	1.89 (0.5)	1.85 (0.5)	1.99 (0.5)	0.001

Left ventricle-to-heart ratio	0.23 (0.05)	0.24 (0.04)	0.26 (0.04)	0.001
Right longitudinal diameter (mm)	17.1(3)	17.0 (2.2)	17.6 (2.1)	0.001
Right basal transverse diameter (mm)	9.9 (1.4)	9.7 (1.3)	10.2 (1)	0.002
Right ventricular sphericity index	1.67 (0.2)	1.69 (0.2)	1.73 (0.2)	0.016
Left longitudinal diameter (mm)	19.7 (3)	19.5 (2)	19.9 (3)	0.004
Left basal transverse diameter (mm)	10.7 (1.5)	10.6 (2)	10.5 (2)	0.001
Left ventricular sphericity index	1.85 (0.2)	1.87 (0.2)	1.90 (0.2)	0.040
Right free diastolic wall thickness (mm)	2.9 (0.4)	2.9 (0.3)	2.6 (0.3)	<0.001
Left free diastolic wall thickness (mm)	2.8 (0.3)	2.9 (0.3)	2.6 (0.3)	<0.001
Septal diastolic wall thickness (mm)	3.0 (0.5)	3.0 (0.4)	2.8 (0.3)	<0.001
Right relative wall thickness (mm)	0.66 (0.14)	0.65 (0.12)	0.56 (0.11)	<0.001
Left relative wall thickness (mm)	0.69 (0.14)	0.67 (0.14)	0.58 (0.12)	<0.001
Systolic function				
Aortic VTI (cm^2)	9.6 (1.8)	9.8 (1.6)	8.1 (1.0)	<0.001
Main pulmonary VTI (cm^2)	9.4 (2)	9.0 (1.5)	8.0 (1.0)	<0.001
Tricuspid annular plane systolic excursion (mm)	6.8 (0.8)	6.9 (0.9)	5.9 (0.7)	<0.001
Mitral annular plane systolic excursion (mm)	4.9 (0.5)	4.9 (0.8)	4.4 (1.1)	<0.001
Isovolumetric contraction time (ms)	31.8 (4)	32.4 (4)	29 (3)	<0.001
Ejection time (ms)	170 (11)	172 (11)	170 (12)	0.966
Myocardial performance index	0.44 (0.06)	0.45 (0.05)	0.40 (0.04)	<0.001
Right stroke volume (mL)	1.84 (0.7)	1.79 (0.5)	1.58 (0.6)	<0.001
Right cardiac output (mL/min)	261 (92)	254 (87)	221 (87)	<0.001
Left stroke volume (mL)	1.72 (0.7)	1.68 (0.5)	1.35 (0.5)	<0.001
Left cardiac output (mL/min)	238 (93)	230 (89)	189 (69)	<0.001
Combined cardiac output (mL/min)	499 (178)	486 (155)	411 (145)	<0.001
Right ejection fraction (%)	79 (5.2)	77 (9.3)	67.2 (11.3)	<0.001
Right shortening fraction	40.2 (5)	42.7 (6,8)	34.4 (8.9)	<0.001

Left ejection fraction (%)	81 (7.5)	79 (8.6)	73.7 (11.2)	<0.001
Left shortening fraction	43.9 (8.4)	42.9 (9.8)	37.2 (9.9)	<0.001
Diastolic function				
Tricuspid E (cm/seg)	37 (8)	38 (8)	34 (7)	0.074
Tricuspid A (cm/seg)	52 (10)	53 (10)	50 (9)	0.062
Tricuspid E/A ratio	0.71 (0.1)	0.70 (0.1)	0.70 (0.1)	0.394
Mitral E (cm/seg)	34 (6)	34 (8)	32 (6)	0.087
Mitral A (cm/seg)	47 (9)	49 (9)	47 (8)	0.374
Mitral E/A ratio	0.72 (0.1)	0.70 (0.1)	0.68 (0.1)	0.044
Isovolumetric relaxation time (ms)	43 (5)	43 (5)	39 (6)	<0.001

Data are mean (SD) or percentage, as appropriate. * p calculated by ANOVA with Bonferroni correction. [ǂ]No significant differences were found between de larger and smaller twin. MCDA: monochorionic diamniotic; PI: pulsatility index, VTI: velocity time integral.

Supplementary Table S2. Sensitivity analysis of the monochorionic and the singleton groups with and without BNP analysis.

	MCDA twin fetuses without BNP (*n*=73)	MCDA twin fetuses with BNP (*n*=27)	*p*
**Doppler**			
Umbilical artery pulsatility index	1.10 (0.2)	1.09 (0.2)	0.525
Middle cerebral artery pulsatility index	1.84 (0.2)	1.85 (0.3)	0.629
Ductus venosus pulsatility index	0.59 (0.2)	0.60 (0.1)	0.537
**Function**			
Isovolumetric contraction time	32 (5.3)	33 (5.0)	0.580
Ejection time	171 (10)	172 (14)	0.465
Isovolumetric relaxation time	43 (4.8)	43 (4.4)	0.421
Myocardial performance index	0.44 (0.05)	0.45 (0.06)	0.524
Right annular plane systolic excursion	6.9 (0.9)	6.9 (0.7)	0.822
Left annular plane systolic excursion	4.9 (0.8)	5.0 (0.7)	0.569
Mitral E/A	0.70 (0.1)	0.71 (0.1)	0.819
Tricuspid E/A	0.72 (0.1)	0.71 (0.1)	0.833
**Morphometry**			

Cardiothoracic ratio	0.26 (0.02)	0.26 (0.03)	0.560
Right atrial area	1.36 (0.3)	1.34(0.3)	0.618
Left ventricular area	1.87 (0.4)	1.89(0.5)	0.440
Left ventricular sphericity index	1.86 (0.2)	1.85 (0.2)	0.421

	Singletons without BNP (*n*=150)	Singletons with BNP (*n*=50)	*p*
**Doppler**			
Umbilical artery pulsatility index	1.02 (0.2)	1.03 (0.2)	0.719
Middle cerebral artery pulsatility index	1.83 (0.3)	1.81 (0.5)	0.510
Ductus venosus pulsatility index	0.54 (0.1)	0.53 (0.1)	0.597
**Function**			
Isovolumetric contraction time	30 (3)	29 (3)	0.558
Ejection time	170 (8)	169 (11)	0.437
Isovolumetric relaxation time	39 (3)	39 (4)	0.411
Myocardial performance index	0.4 (0.03)	0.4 (0.04)	0.562
Right annular plane systolic excursion	5.8 (0.6)	5.9 (0.7)	0.498
Left annular plane systolic excursion	4.4 (0.5)	4.5 (0.7)	0.420
Mitral E/A	0.69 (0.1)	0.68 (0.1)	0.787
Tricuspid E/A	0.70 (0.1)	0.70 (0.1)	0.654
**Morphometry**			
Cardiothoracic ratio	0.26 (0.03)	0.26 (0.03)	0.530
Right atrial area	1.20 (0.3)	1.22 (0.4)	0.444
Left ventricular area	1.98 (0.3)	2.0 (0.4)	0.504
Left ventricular sphericity index	1.89 (0.3)	1.91 (0.3)	0.630

Data are mean (SD); BNP: B-type natriuretic peptide; MCDA: Monochorionic diamniotic.
